# Development and validation of an instrument to measure physician awareness of bioethics and medical law in Oman

**DOI:** 10.1186/s12910-021-00619-1

**Published:** 2021-05-22

**Authors:** Ahmed S. Al-Busaidi, Anuradha Ganesh, Samir Al-Adawi, Yahya M. Al-Farsi, Maryam K. Al-Rawahi, Nusaiba A. Al-Mawali, Nadiya S. Al-Kharousi, Mohammed Al-Alawi, Abdullah S. Al-Mujaini

**Affiliations:** 1 Directorate General of Primary Health Care, Ministry of Health, Muscat, Sultanate of Oman; 2 Department of Ophthalmology, College of Medicine and Health Sciences, Sultan Qaboos University, Muscat, Sultanate of Oman; 3 Department of Behavioral Medicine, College of Medicine and Health Sciences, Muscat, Sultanate of Oman; 4 Department of Family Medicine and Public Health, Sultan Qaboos University, Muscat, Sultanate of Oman; 5 Medical Student, College of Medicine and Health Sciences, Sultan Qaboos University, Muscat, Sultanate of Oman

**Keywords:** Medical ethics, Cross-sectional study, Medical law, Oman, Questionnaire, OBMLA

## Abstract

**Background:**

A different ethos with respect to the perception of medical ethics prevails in societies in transition such as those in the Arabian Peninsula, which makes it difficult to apply international principles of bioethics in medical practice. This study aimed to develop and psychometrically test an instrument that measures physicians’ awareness of bioethics and medical law and their attitudes towards the practice of medical ethics. Additionally, it examined physician correlates influencing the awareness of bioethics.

**Methods:**

Following a rigorous review of relevant literature by a panel of experts, a 13-item instrument, the Omani physicians’ bioethics and medical law awareness (OBMLA) questionnaire was developed with the aim of assessing physicians’ awareness of bioethics and medical law. The study tool’s construct validity and internal consistency reliability were examined by exploratory factor analysis (EFA) and Cronbach’s alpha. In a cross-sectional study, the questionnaire was distributed among a random sample of 200 physicians at a tertiary hospital in Muscat, Oman. Participant characteristics that may influence awareness of bioethics and medical law were explored.

**Results:**

The EFA of the OBMLA questionnaire resulted in three well-loading factors: (1) Physicians’ bioethics practice subscale (2) incentive related bioethics subscale and (3) medical law awareness subscale. Internal consistency reliability ranged between Cronbach’s α: 0.73–0.8. Of the total 200 participants, 52% reported that teaching medical ethics during medical school was inadequate. The overall mean (standard deviation, SD) of the bioethics awareness score and Omani medical law awareness were 27.6 (3.5) and 10.1 (2.1) respectively. The majority of physicians (73%) reported that they frequently encountered ethical dilemmas in their practice and 24.5% endorsed the view that unethical decisions tended to occur in their practice.

**Conclusion:**

The study provides an insight into the practice of bioethics, and the awareness of bioethics and medical law among physicians in a teaching hospital in Oman. The OBMLA questionnaire appears to be a valid and reliable tool to assess a physician’s awareness of bioethics and medical law. In this preliminary study, it appears that participants have suboptimal scores on the indices which measure practice and awareness of bioethics and medical law.

**Supplementary Information:**

The online version contains supplementary material available at 10.1186/s12910-021-00619-1.

## Background

Physicians routinely encounter ethical dilemmas pertaining to matters such as informed consent, patient confidentiality, deceptions and non-disclosure, determination of death, doctor-patient relationship, acceptance of gifts from drug companies, sexual contact between physicians and clients, and misconduct related to research and publication. Such ethical dilemmas are increasingly addressed by the emerging field of medical ethics also known as bioethics [1].

Medical ethics refers to “the analytical subject in which concepts, assumptions, beliefs, attitudes, emotions, reasons and arguments underlying medico-moral decision making are examined critically” [2]. Respect for autonomy, beneficence, non-maleficence and justice are four key principles of biomedical ethics [3]. Medical ethics guides physicians in their relationships with patients, patients' families and colleagues. These defined behaviors are considered mandatory and aid decision-making in medical practice, education and research [4]. The instrumental role of bioethics has been consolidated in 2005 during the 33rd session of the General Conference of UNESCO when the *Universal Declaration on Bioethics and Human Rights* was adopted [5]. UNESCO’s document addresses moral discernment relating to medical policy, practice and professionalism as well as controversial ethical issues emerging from new situations and possibilities brought about by advances in biomedical sciences [6].

Despite its universal appeal, the Universal Declaration on Bioethics and Human Rights has not been heeded in many societies in transition (non-western societies), where tradition pervades all dimensions of life [7]. Traditional ethos may supersede the principles of bioethics in such societies and they may not readily embrace existing principles of bioethics. It has been suggested that the patient's ethos renders medical practice to be congruent with good practice in bioethics [8–10]. However, studies on awareness of the principles of bioethics and medical law in societies transition such as Nepal [11], India [12], the Caribbean [13], Africa [14, 15], and the Middle East [16] report that the practice of the code of ethics among medical practitioners in these countries leaves a lot to be desired, and have recommended education as an antidote to laxity in the application of principles of bioethics.

The current study has two interrelated objectives. The first is to develop and validate an instrument to assess the physician’s awareness of medical ethics and related policy in Oman, an Islamic country located in the southern tip of the Arabian Peninsula. The second objective is to investigate participant characteristics associated with higher awareness scores.

## Methods

### Setting, duration, design

A cross-sectional study was conducted between March and June 2016 among physicians working at the Sultan Qaboos University Hospital (SQUH), Muscat, Oman. SQUH is a tertiary referral center that was established under the auspices of Oman’s Ministry of Higher Education in 1990. The mandate of SQUH includes the provision of medical education and medical care to the country. Physicians working at SQUH are of diverse nationalities; enrolled students are graduates or postgraduates of Omani nationality.

### Participants and sampling method

The participants of this study consisted of practicing physicians of different nationalities and rank rotating in SQUH (interns, residents, senior house officers, registrars, senior registrars, consultants, and senior consultants) belonging to various clinical specialties (diagnostic, medical and surgical units). A prerequisite for participating in the study was that the physician should have regular contact with patients. The study was stratified, based on rank, using a simple random sampling method to recruit the study participants.

### Sample size

Sample size estimation was performed by OpenEpi software. Considering the total number of physicians working at the hospital during the study period, an 80% power, 5% level of type 1 error, and an a priori estimate that 40% of physicians would have high knowledge of bioethics [17], the required sample size was found to be 210.

### Data collection process

The questionnaires were distributed by trained medical students who explained the importance of the study and motivated the participants.

### Instrument development

#### Step 1: Design of the questionnaire

A focus group comprising of an academic professor, two consultant physicians who are experts in the field of medical ethics and an expert in Oman Medical law and policy, worked to decipher the themes related to a theoretical framework and construction of the questionnaire items after extensive literature review [18–22]. As a culture-specific instrument for tapping the awareness of bioethics is still nascent, the present questionnaire was developed in English, with the rationale that English is a ‘lingua franca’ of the medical, nursing and allied health education in Oman. Proficiency in English is a prerequisite for enrolment in medical education in Oman [23].

After careful consideration, items from relevant published work in this area were appraised and modified. A 13-item instrument was formulated. The designed questionnaire aimed to cover two related constructs, namely the physician’s awareness and practice of principles of bioethics and the physician’s awareness of Omani medical laws governing medical practice. In addition, the questionnaire included questions related to the socio-demographic characteristics of the participant, the teaching of medical ethics at medical school and details of ethical encounters (Additional file 1).

### The content of the questionnaire

The items (1 to 8) in section C of the questionnaire were to be answered with a four-point Likert scale type of response: ‘Ethical’ (1), ‘Uncertain’ (2), ‘Somewhat unethical’ (3), and ‘Unethical’ (4). Items (9 to 13) in section D pertained to the awareness of Omani medical laws [22], and required responses on a 3-point Likert scale: ‘Legal’ (3), ‘Uncertain’ (2) and ‘Illegal’ (1).

#### Step 2: Testing the face and content validity

Face and content validity were evaluated independently by a panel of international experts in medical law and bioethics, physicians and patients. Three items were deemed redundant and removed after discussion between the study panel and the independent panel. After the process of revision, a 13-item draft questionnaire (8 items related to the ethical practice and 5 items related to the Omani Medical laws) was developed.

#### Step 3: Examining construct validity and reliability

The construct validity was assessed using exploratory factor analysis (EFA) using principal axis factoring as the extraction method and varimax rotation with Kaiser normalization. To test the internal consistency of the questionnaire, Cronbach’s α coefficient was utilized. Correlations between factors were assessed with Pearson’s correlation.

### Statistical analysis

Data analysis was performed with SPSS software version 22. The socio-demographic factors were presented as frequency and percentages. Chi-square analysis was used to evaluate the statistical significance of differences among proportions of categorical data. The non-parametric Fishers’ exact test (two-tailed), instead of the Chi-square test was used for small sample sizes, where the expected frequency was less than 5 in any of the 2 × 2 table cells.

The questionnaire’s total score and subscales scores normality of distribution were examined with Kolmogorov–Smirnov and Shapiro–Wilk normality test. Means and standard deviations of the same were also reported.

For EFA, factors with eigenvalues of more than 1 were selected according to Kaiser’s criterion. Item loading of more than 0.4 was set to for items inclusion under certain factor. Bartlett's test and Kaiser–Meyer–Olkin test assessed the suitability of our data for factor analysis. At the univariate analysis level, independent sample T-test and ANOVA examined associations between socio-demographic variables and questionnaire mean scores. Homogeneity of the variance between the groups was tested by Levene’s Test for Equality of Variances before running ANOVA.

Multivariate, linear regression analysis was employed to predict the mean scores of subscales of the study tool from the associated factors at univariate analysis. In the regression model, the Enter method was chosen with entry and removal criteria as follows: *P* value 0.05 and 0.1 respectively. Model fitness was evaluated with the F test and R squared. Beta coefficients were reported. A *P* value of less than or equal to 0.05 was considered as the level of significance.

## Results

### Instrument development—validity, reliability and correlation

**Fig. 1:**
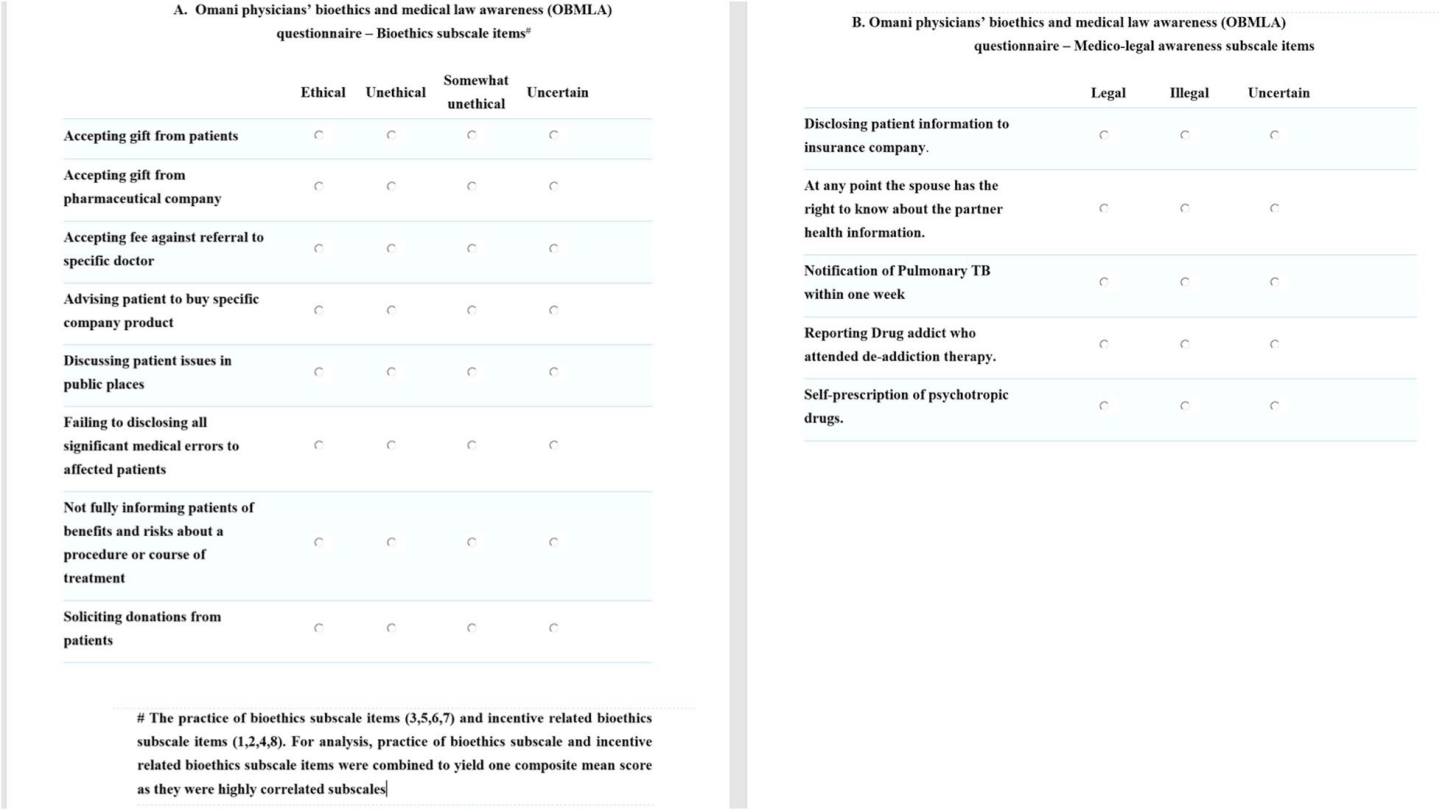
Oman Physician’s Bioethics and Medical Law Awareness (OBMLA) questionnaire with three factors with an eigenvalue of more than 1 - ‘physician ethical practice’ subscale (items 1, 2, 4, 8), ‘patient ethics-related’ subscale (items 3, 5, 6, 7) and ‘Oman medical law subscale’ (items 9–13)

Exploratory factor analysis (EFA) resulted in the three factors with an eigenvalue of more than 1 (Fig. 1). Two factors were related to physician ethical practice (incentive related subscale—items 1, 2, 4, 8, and patient ethics related subscale—items 3, 5, 6, 7). One factor was related to Oman medical law (items 9–13). The items were loading well in each main factor. The internal reliability (Cronbach’s alpha) of the three factors were 0.73, 0.8 and 0.74 respectively. The correlation between the two factors related to physician awareness of the principles of bioethics and the awareness of Omani Medical laws was high (Pearson r = 0.8). However, a low correlation (Pearson r = 0.4) was observed between the factor (Oman Medical law and policy subscale) and the other two factors.

Table 1 shows the general characteristics of the study participants. Out of the 210 participants recruited for the study, 10 eligible participants were excluded due to lack of response and incomplete questionnaires. A total of 200 physicians were enrolled; 107 (53.5%) of them were males and 93 (46.5%) were females. More than half of the participants studied medicine in Oman (58%). A total of 51% had junior clinical rank designation, 45.5% were aged between 25 to 30 years, and 42.5% had less than five years of experience. The proportion of participants who studied medicine in countries other than Oman was significantly higher among females (49.5% vs. 33.3%; *P* = 0.02).

**Table 1:** Characteristics of study participants by gender, Oman, 2016

Characteristics	Total (N = 200)
	N (%)
*Gender*	
Male	107 (54)
Female	93 (46)
*Age*	
25 to 30	91 (45.5)
31 to 40	53 (26.5)
Above 40	56 (28.0)
*Rank designation*	
Senior House Officer	102 (51.0)
Registrar	54 (27.0)
Consultant	44 (22.0)
*Experience (years)*	
Less than 5	85 (42.5)
5 to 10	39 (19.5)
Above 10	76 (38.0)
*Specialty*	
Surgical	71 (35.5)
Medical	129 (64.5)
*Place of studying medicine*	
Oman	116 (58.0)
Elsewhere	84 (42.0)
*Regularly work with patients*	
No	4 (2.0)
Yes	196 (98.0)

Table 2 shows the indices of teaching and knowledge of bioethics. Overall, 70.5% of the students reported that they had 1 to 5 credit hours of teaching ethics in their medical school curriculum. More than half (52%) of the students reported that the teaching to be inadequate and that the learning resources were insufficient. Strikingly, 70% of the students reported that the ethics principles learned in medical school had weak relevance to medical practice. Of the participants, 65% reported that ethics can be taught in medical schools and 41% reported a need for more ethics teaching in medical school, and 21% believed that ethics cannot be taught in medical schools. Overall, 86% affirmed that they knew the four basic principles of ethics.

**Table 2:** Awareness bioethics/issue pertinent to teaching of bioethics (n = 200) at the teaching hospital in Oman 2016

	Characteristics	Total
		(N = 200)
		N (%)
During medical school, how many credit hours were there for ethics in your curriculum?	1 to 5	141 (70.5)
	6 to 10	40 (20.0)
	> 10	19 (9.5)
Do you think that the teaching about medical ethics in medical school was adequate?	Yes	77 (38.5)
	No	105 (52.5)
	Don’t know	18 (9.0)
Do you think that the resources provided for teaching of medical ethics in your medical school were sufficient?	Yes	57 (28.5)
	No	113 (56.5)
	Don’t know	30 (15.0)
How do you rate the relevance of the teaching of medical ethics to your practice now?	Strong	59 (29.5)
	Weak	141 (70.5)
Do you think that you need more of teaching of medical ethics?	Yes	83 (41.5)
	No	89 (44.5)
	Don’t know	28 (14.0)
Do you think that medical ethics can be taught?	Yes	131 (65.5)
	No	42 (21.0)
	Don’t know	27 (13.5)
Do you know the four principles of medical ethics?	Yes	172 (86.0)
	No	15 (7.5)
	Don’t know	13 (6.5)

Table 3 shows the indices of practicing medical ethics in professional life among study participants. The majority (73%) reported frequent encounters of ethical dilemmas in their professional practice, and 24.5% reported frequent observation of an unethical decision being made. The most frequently reported areas where ethical dilemmas were encountered involved traditions and values, followed by religion and conflict of interest, while the least reported areas were in law and finance.

**Table 3:** Practice of bioethics among physicians (n = 200) at the teaching hospital in Oman 2016

	Characteristics^a^	Total (N = 200)
		N (%)
How often do you encounter an ethical situation in your practice?	Rare	12 (6.0)
	Occasional	42 (21.0)
	Often	67 (33.5)
	Frequent	79 (39.5)
How often you observe an unethical decision in your practice?	Rare	77 (38.5)
	Occasional	74 (37.0)
	Often	34 (17.0)
	Frequent	15 (7.5)
	Rare	18 (9.0)
How often do you find an answer to your ethical dilemma?	Occasional	76 (38.0)
	Often	71 (35.5)
	Frequent	34 (17.0)
In what area do you encounter ethical issues?	Religion	106 (53.0)
	Law	73 (36.5)
	Finance	65 (32.5)
	Conflict of interest	101 (50.5)
	Traditions and values	121 (60.5)
Where usually you look for an answer for your ethical question?	Books	53 (26.5)
	Internet	99 (49.5)
	Friends	35 (17.5)
	Senior colleague	156 (78.0)
	Elsewhere	19 (9.5)
What mostly stops you from unethical practice?	Religion	130 (65.0)
	Traditions and values	109 (54.5)
	Law	87 (43.5)
	Ethics teaching	99 (49.5)

^a^Rare = once a year; Occasional = once every six months; Often = once every month; Frequent = once every week

The most common sources where physicians obtained solutions to ethical dilemmas were senior colleagues (78%), followed by the internet (49%) and books (26%), while the least reported sources were friends (17%) and other sources. Only 52.5% reported they found answers to the ethical dilemmas in a satisfactory manner. The proportion of the participants who reported an inability to find answers to ethical dilemmas was as high as 47%. The most influential factors that deter physicians from unethical practice were their religious background (65%), tradition and values (54%), and ethics teaching (49%), and the least influential deterrent was existing law (43%).

Table 4 shows the means and standard deviations of the bioethics awareness score and the Omani medical law awareness score among participating physicians over selected socio-demographic and professional participant characteristics. The overall mean (standard deviation, SD) of the bioethics awareness score was 27.6 (3.5). The score was slightly higher among males when compared to females but the difference was not statistically significant (27.7 vs. 27.5; *P* = 0.72). The mean bioethics awareness score was higher with increasing age, rank, and years of experience (*P* < 0.05). The score was slightly higher among physicians specializing in surgery when compared to physicians of other medical specialties. The scores were also found to be significantly higher (*P* = 0.001) among those who studied medicine outside Oman.

**Table 4:** Indices of bioethics awareness and Medico-legal awareness scores (mean, SD) among Omani physicians by selected socio-demographic and professional characteristics, Oman, 2016

Characteristics	Bioethics awareness (practice of bioethics and incentive related bioethics subscales)		Medico-legal awareness subscale	
	Mean (SD)	*P* value	Mean (SD)	*P* value
*Overall*	27.6 (3.5)		10.1 (2.1)	
*Gender*		0.72		0.15
Male	27.7 (3.9)		10.2 (2.0)	
Female	27.5 (2.9)		9.4 (2.1)	
*Age*		0.001		0.67
25 to 30	26.6 (3.7)		9.9 (1.8)	
31 to 40	28.3 (3.1)		10.3 (2.2)	
Above 40	28.5 (3.0)		9.9 (2.3)	
*Rank designation*		0.001		0.86
Senior House Officer	26.7 (3.7)		10.0 (1.8)	
Registrar	28.9 (2.6)		10.1 (2.5)	
Consultant	28.0 (3.4)		10.2 (1.9)	
*Experience (years)*		0.002		0.83
Less than 5	26.7 (3.8)		10.1 (1.8)	
5 to 10	27.0 (2.9)		9.9 (2.1)	
Above 10	28.8 (2.9)		10.1 (2.3)	
*Specialty*		0.35		0.85
Surgical	27.2 (4.1)		10.0 (2.2)	
Medical	27.8 (3.1)		10.1 (2.0)	
*Place of studying medicine*		0.001		0.43
Oman	26.8 (3.7)		10.1 (1.8)	
Elsewhere	28.6 (2.8)		9.9 (2.3)	
*Regularly work with patients*		0.008		0.02
No	27.7 (2.1)		12.5 (1.2)	
Yes	27.1 (3.5)		10.0 (2.1)	

The overall mean (SD) of the Omani medical law awareness score was 10.1 (2.1). The score was slightly higher among males when compared to females but the difference was not statistically significant (10.2 vs. 9.4; *P* = 0.15). The mean medical law awareness score had variant trend with the increase in age, rank designation, and years of experience and the differences were not statistically significant (*P* < 0.05). The score was slightly higher among physicians in medical compared to surgical specialty, and among those who studied medicine in Oman.

Table 5 shows multi-linear regression model of the association between the bioethics awareness score and the Omani medical law awareness score with selected socio-demographic and professional characteristics of participants. Years of experience and place of studying medicine were found to be the major predictors of the bioethics awareness score after adjusting for other factors, but the associations were not significant (β = 0.170 and β = 0.158 respectively, *P* > 0.05). Other predictors had little influence on the bioethics awareness score and were not statistically significant.

**Table 5:** Multivariate linear regression modelling of the association between the bioethics awareness and medico-legal scores with selected socio-demographic and professional characteristics of participants, Oman, 2016

Predictor	Bioethics awareness (practice of bioethics and incentive related bioethics subscales) score			Medico-legal awareness subscale score		
	β	SE	*P*	β	SE	*p*
Gender	0.037	0.507	0.61	− 0.137	0.306	0.063
Age	0.004	0.663	0.98	− 0.007	0.402	0.965
Rank designation	0.001	0.455	0.99	0.02	0.276	0.851
Experience	0.170	0.634	0.29	0.045	0.384	0.787
Specialty	0.071	0.511	0.31	0.007	0.309	0.919
Place of studying medicine	0.158	0.661	0.11	0.007	0.309	0.919

β refers to standardized β; SE refers to standard error; *P* refers to *P* value

## Discussion

Medical schools and accompanying teaching hospitals have proliferated in the Arabian Gulf countries including Oman in the past decade. Healthcare infrastructure in Oman has been internationally lauded for its efficiency [24]. Medical ‘culture’ and the required awareness of medical ethics are increasingly recognized as an inalienable part of medical practice and intimately linked to medical professionalism [5]. It is considered as best practice in medical settings [5]. Physician awareness of the principles of bioethics and medical law in such healthcare systems in the Arabian Gulf countries remains largely unanswered. A cross-sectional study was conducted to explore the practice and awareness of bioethics among physicians working in a teaching hospital in Oman.

For exploring the physician awareness and the practice of bioethics and medical law, an instrument entitled the Oman physician’s bioethics and medical law awareness (OBMLA) questionnaire was designed for physicians practicing in Oman. Exploratory factor analysis (EFA) generated three factors: (1) the practice of bioethics subscale items, (2) incentive related bioethics subscale items, and (3) medico-legal awareness subscale items. The internal consistency and reliability of the OBMLA questionnaire were found to be adequate.

Oman has one governmental (public) university for teaching medicine and one private medical school. A teaching hospital is a vanguard for inoculating tomorrow’s doctors with the essence of medical professionalism. This study targeted physicians of all specialties, designations, and nationalities. The study sample thus represented the cadres of physicians in Oman.

A laxity in the awareness of bioethics and medical law was perceived among physicians in Oman. The current study combined many variables to answer the question as to what factors influenced the practice of ethical medicine. Through regression analysis, more specifically, multivariate linear regression modelling, the association between awareness of bioethics and Omani medical law scores with selected socio-demographic and professional characteristics of participants were examined.

Waddell [25] noted that it is not clinical issues but individual characteristics that shape medical professionalism. Previous studies have identified many attributes among physicians that vary between genders including emotional intelligence [26], physician–patient relationship [27], and indices of professionalism [28]. The influence of gender on the practice of bioethics was analyzed. Male physicians appeared more likely to be faced with ethical dilemmas. The gender gap was statistically significant in ethical dilemmas pertaining to law and finance. Although it did not reach statistical significance, genders also differed in their response to conflicts of interest.

A majority of participants endorsed that they had minimal exposure to the education of bioethics. The teaching of bioethics in medical school was perceived to be inadequate in terms of content and number of hours assigned for the course by most of the participants. In this study, senior doctors were more likely to have obtained their medical degrees from outside Oman, and junior doctors were more likely to be qualified from Oman. Participants who had obtained their medical degree from outside Oman were found to have more hours of exposure to the teaching of bioethics and therefore had better knowledge of bioethics.

In general, the teaching of bioethics in medical schools in Western Europe and North American is highly developed [29], in contrast to non-western societies [30, 31]. Alkabba et al. [17] evaluated teaching of bioethics in 14 medical schools in Saudi Arabia and reported that while medical schools did offer courses in bioethics, none of the schools had a dedicated unit for teaching the same and the teaching of bioethics was sporadic and substandard. Lehmann et al. [29] surveyed medical ethics education in US and Canadian medical schools and suggested that insufficient time allocated in the curriculum for teaching bioethics and dearth of competent teachers were the main obstacles to the implementation of ethics education among students. Capacity development is essential, if not paramount, for the development of professionalism linked to bioethics in the Arabian Gulf countries. The content of the medical ethics curriculum, the number of hours dedicated to them, and the approach towards teaching needs to be standardized [29, 32]. In Saudi Arabia, Alkabba et al. [17] have called for the standardization of the teaching of bioethics in medical schools through the introduction of interactive and student-engaging methods as opposed to passive lecturing. In Oman, the recently introduced Oman Medical Specialty Board that supervises resident training in the country, has embraced accreditation by the Accreditation Council for Graduate Medical Education–International and has a strong focus on bioethics in its curriculum [23]. How the new curriculum would salvage the present suboptimal literacy and the awareness of bioethics and medical law remains to be seen.

The study revealed that physicians frequently encounter ethical dilemmas and unethical practices during routine work and feel ill-equipped to deal with them. In an attempt to resolve these dilemmas, they sought help from their senior colleagues or searched the internet or available literature. This scenario has been documented in other studies as well [33]. The endorsed unethical practices in this study are likely to have negative repercussions. Al-Mandhari et al. [34] conducted a community survey to assess the understanding of the term “medical error” among the Omani general public. The study indicated that 49% were aware of what constitutes a medical error and 49% felt the primary cause of a medical error was suboptimal professionalism among healthcare workers. This negative perception has been speculated to contribute towards many Omanis seeking healthcare services outside Oman [35] even though Oman has been lauded to have one of the most efficient healthcare systems in the world [24].

In this study, physicians reported that the majority of issues that posed ethical dilemmas were those that clashed with the prevalent social and cultural practices or spiritual teaching. The study highlighted the most influential factor that influenced the participants from abstaining from unethical practices to be religion and traditional values. Alkabba et al. [17] listed ten major ethical issues perceived by physicians in Saudi Arabia including patients' rights, equity of resource distribution, patient confidentiality, patient safety, conflicts of interest, ethics of privatization, informed consent, dealing with the opposite gender, beginning and end of life issues, and healthcare team ethics, and argued that very little attention has been given to these challenges in Saudi Arabia. They called for the initiation of more in-depth discussions on the ethical issues, to bring about changes in policies, particularly on resource allocation. Some of the issues raised by Alkabba et al. [17] were applicable to physicians in Oman; this is understandable as Oman shares the Arabic-Islamic moral values with Saudi Arabia.

Ethical dilemmas often fall into two broad categories. One with affinity to moral discernment which, in turn, defines medical policy, practice and professionalism. The second category comprises situations that have arisen due to the emergent and new practices in biomedical sciences. Regardless of the type, little research has explored the suitability of implementing practices in a society where the ethos of life is different from the tenet of modern ethical principles [17]. Existing principles of bioethics rest strongly on the western philosophical principles of respect for persons and a strong emphasis on autonomy [36]. In collective societies in the Arabian Peninsula, commonly considered as societies in transition, such an ethos appears to be alien and therefore deemed to be a source of attrition rather than suitable practice. Medical practitioners practice medicine without adhering to international bioethical standards stipulated by UNESCO [37]. There are some strong critiques of those who enforce western principles of bioethics without considering the organic sociological bond of the society [38]. At the same time, for accreditation by international bodies, medical schools should have an internationally accepted curriculum of bioethics. Recognizing the diversity of cultures and what might be considered as the norm in certain societies, principles of bioethics may need to take into consideration Islamic law, which focuses on duties and obligations as delineated in the Qur'an or the teachings of Prophet Muhammed [39]. The current study plays an important role in examining the suitability of existing bioethics principles in a non-western population. Related to this, participants drawn for this study have been trained in different parts of the world (42% were trained outside Oman). Oman’s healthcare system comprises of workers from different parts of the world. Indeed, the bulk of health-care workers in Oman are likely to be contract workers from different parts of the world. A concerted effort to implement secular or ‘culture-free’ bioethics is needed in Oman. Amid globalization, medical curriculum developers in Oman need to be cognizant of the need for pluralism in bioethics.

This study also explored physician awareness of Omani medical law and the code of professional conduct for doctors. Majority of participants were uncertain of some of the stipulations in the Omani Medical law. Improved awareness is anticipated to improve the relationship of physicians with patients and their families. This would in turn maintain a high and healthy trust in the healthcare system by the general public of Oman, as found by the survey conducted in 2012 among members of the general public in Oman to explore the preferences for and the perceptions of medical error disclosure (MED) by the public [40]. The study revealed a disclosure gap between the respondents’ preferences for MED and the perceived current MED practices in Oman. It called for addressing this issue to increase public confidence in the national healthcare system. With respect to patient confidentiality, again a majority of practitioners were uncertain about the Omani medical law; similar misunderstandings persisted with respect to the law regarding substance misuse and self-prescription of psychotropic medications. On the contrary, a majority of participants were aware of what to do with a patient with pulmonary tuberculosis. According to the code of conduct, physicians should alert the communicable disease surveillance and control section under the auspices of the Ministry of Health. Confidentiality is waived if the patient has a condition that could affect public health [1]. There is a need to heighten awareness of Omani Medical Laws amongst doctors in Oman.

### Limitations

This study is not without limitations. First, the non-probability sampling method (convenience sampling) was used to collect the data from physicians at one hospital and hence results cannot be generalized to the whole country. In order to scrutinize the findings from this study, the study would have to be extended to other healthcare settings in Oman. Second, the relatively small sample size affected the power of the study to detect differences. Not all observations in this study were statistically significant across the categories. Thirdly, the study was cross-sectional, so the observed trend does not postulate the cause and effect due to a lack of temporality and potentially reversed causality in cross-sectional studies. The newly developed OBMLA questionnaire was scrutinized for construct validation. Future studies could employ other instruments for construct validation. Considering the significance of interface between culture or religion, a wider group for the development of the study questionnaire, with inclusion of scholars in cultural anthropology and religion might be desirable. We recommend future refinement of OBMLA questionnaire with supplementation of this scale with a few specific questions addressing religious and cultural issues. Finally, the study would have more generalizability if it employed a previously validated instrument to study awareness and practice of medical ethics. However, extensive literature search did not reveal the existence of such an instrument.

## Conclusion

This study embarked with two aims. The first was to describe an instrument for identifying the awareness of principles of bioethics and medical law amongst physicians in Oman. This exercise led to the development of the Oman physician’s bioethics and medical law awareness (OBMLA) questionnaire. Exploratory factor analysis resulted in the three factors with adequate psychometric properties. The follow-up cross-sectional survey using the OBMLA questionnaire suggests that ethical issues are frequently encountered by physicians in a teaching hospital in Oman. Many physicians are unaware of ethical and legal issues relevant to medical practice in the country. The current state of ethics education is perceived to be inadequate and does not ensure a common standard for the practice of medical ethics and the awareness of medico-legal issues. The study calls for more attention to be directed towards the content and the duration of ethics education in the medical curriculum. It also identified a necessity for ethics learning to be periodically reinforced through continuing medical education programs and forums providing ongoing supervision, guidance and support to physicians facing ethical dilemmas. There is also a need to contemplate an ethics teaching program that is culturally sensitive.

## Supplementary Information

**Additional file 1**. A 13 items instrument. The designed questionnaire aimed to cover two related constructs, (a) awareness and practice of principles of bioethics and (b) awareness of Omani medical laws governing medical practice. In addition, the questionnaire included questions related to socio-demographic characteristics of the participant, the teaching of medical ethics at medical school and details of ethical encounters.

## Data Availability

This is a research article and all data generated or analyzed during this study are included in this published article.

## Abbreviations

EFA: Exploratory factor analysis
MED: Medical error disclosure
OBMLA: Omani physicians’ bioethics and medical law awareness
SD: Standard deviation
SQUH: Sultan Qaboos University Hospital
UNESCO: United Nations Educational, Scientific and Cultural Organization
