# Supplementary material for: Development and validation of an instrument to measure physician awareness of bioethics and medical law in Oman
Source: BMC Med Ethics. 2021 May 22;22:65. doi: 10.1186/s12910-021-00619-1 (PMC8140473; doi:10.1186/s12910-021-00619-1)
Supplement: Supplementary file 1 — Additional file 1. A 13 items instrument. The designed questionnaire aimed to cover two related constructs, (a) awareness and practice of principles of bioethics and (b) awareness of Omani medical laws governing medical practice. In addition, the questionnaire included questions related to socio-demographic characteristics of the participant, the teaching of medical ethics at medical school and details of ethical encounters. [file 12910_2021_619_MOESM1_ESM.docx]

**Perception of doctors about Medical Ethics**

**Practice and Teaching**

**Dear Doctor, This survey on Medical Ethics is being conducted by the Faculty of Sultan Qaboos University, Oman. The survey asks for your views and experience as a physician who currently provides direct patient care, regarding the practice and teaching of Ethics in Medicine. This study is approved by the Medical Research Ethics Committees in the College of Medicine, Sultan Qaboos University. Thank you very much for your participation.**

1. **About you**
2. **Please select your gender?**  Male  Female
3. **Please indicate your age?** 25 – 30 31 – 40 > 40
4. **Do you regularly work with patients at the hospital?** Yes No
5. **Select the area of your post?**

Intern Resident; year…. Senior house office Registrar

Sr. Registrar Consultant and above Other

1. **Where did you study medicine? ( MD Degree )** Oman Elsewhere

1. **How long have you been in practice?**  < 5 Years 5 - 10Years > 10 Years

**If you have received training in medical ethics during medical school, please answer questions 7 to 14, if not go to question 15**

1. **During medical school, how many credits hours were there for ethics in your curriculum?**

1 - 5 Hours 6 - 10 Hour > 10 Hours

1. **Do you think that the teaching about medical ethics in medical school was adequate?**

Yes No I don't know

1. **Do you think that the resources provided for the teaching of medical ethics in your medical school were sufficient?**

Yes No I don't know

1. **How do you rate the relevance of the teaching of medical ethics to your practice now?**

|  | 1 | 2 | 3 | 4 | 5 | 6 | 7 | 8 | 9 | 10 |  |
| --- | --- | --- | --- | --- | --- | --- | --- | --- | --- | --- | --- |
| not relevant at all |  |  |  |  |  |  |  |  |  |  | Very relevant |

1. **Do you think that the amount of teaching you had, helps identify and cope with ethical issues now?**

Yes No  I don't know

1. **Do you think that you need more teaching of medical ethics?**

Yes No I don't know

1. **Do you think that medical ethics can be taught?**

Yes No I don't know

1. **Do you know the four principles of medical ethics?**

Yes No

1. **Ethics in Practice**
2. **How often do you encounter an ethical situation in your practice?**

Rare (once a year) Occasional (once every 6 months)

Often (once every month) Frequent (once every week)

1. **How often do you find an answer to your question on the ethical issue?**

Rare (<25% of the time) Occasional (25-50-% of the time)

Often (50-75% of the time) Frequent (>75% of the time)

1. **How often you observe an unethical decision in your practice?**

Rare (once a year) Occasional (once every 6 months)

Often (once Every Month) Frequent (once every week)

1. **In what area do you encounter ethical issues? Check all that applies to your practice**

Religion Law Financial

Conflict of interest Traditions and Value

Other……………….. ………………………….

1. **Where usually you look for an answer for your ethical question? Check all that applies to your practice**

Books Internet Friends

Senior colleague Elsewhere…………………………………..

1. **What mostly stops you from unethical practice? Check all that applies to you**

Religion Traditional and values Law

Ethics teaching Other………………………………………..

1. **Specific issues in medical ethics**
2. **For the following scenarios select the answer that you feel is most appropriate**

|  |  | **Ethical** | **Unethical** | **Somewhat unethical** | **Uncertain** |  |
| --- | --- | --- | --- | --- | --- | --- |
| **Accepting a gift from patients** |  |  |  |  |  |  |
| **Accepting a gift from pharmaceutical company** |  |  |  |  |  |  |
| **Accepting fee against referral to a specific doctor** |  |  |  |  |  |  |
| **Advising patient to buy specific company product** |  |  |  |  |  |  |
| **Discussing patient issues in public places** |  |  |  |  |  |  |
| **Failing to disclose all significant medical errors to affected patients** |  |  |  |  |  |  |
| **Not fully informing patients of benefits and risks about a procedure or course of treatment** |  |  |  |  |  |  |
| **Soliciting donations from patients** |  |  |  |  |  |  |

1. **Specific issues in Omani medical Law**
2. **For the followings please indicate whether it is legal/illegal or Uncertain according to Omani Law**

|  |  | **Legal** | **Illegal** | **Uncertain** |  |
| --- | --- | --- | --- | --- | --- |
| **Disclosing patient information to the insurance company**. |  |  |  |  |  |
| **Sharing the partner's health information with the spouse .** |  |  |  |  |  |
| **Notification of Pulmonary TB within one week** |  |  |  |  |  |
| **Reporting a drug addict who attended de-addiction therapy.** |  |  |  |  |  |
| **Self-prescription of psychotropic drugs.** |  |  |  |  |  |

**Questionnaires by their nature are limited. Please write in any comments you have about important issues facing ethics in medicine. Thank you very much for your participation in this survey.**

-------------------------------------------------------------------------------------------------------------------------------------------------

-------------------------------------------------------------------------------------------------------------------------------------------------

-------------------------------------------------------------------------------------------------------------------------------------------------

-------------------------------------------------------------------------------------------------------------------------------------------------

-------------------------------------------------------------------------------------------------------------------------------------------------
